# Editorial: Reviews in hepatobiliary diseases 2023

**DOI:** 10.3389/fmed.2024.1468778

**Published:** 2024-09-03

**Authors:** Xin Quan, Lu Yu, Huan Tong

**Affiliations:** Department of Gastroenterology and Hepatology, West China Hospital, Sichuan University, Chengdu, China

**Keywords:** liver cirrhosis, non-alcoholic fatty liver disease (NAFLD), alcoholic liver disease, sphincter of Oddi dysfunction, endoscopic retrograde cholangiopancreatography (ERCP), liver surgery

Hepatobiliary diseases currently affect an enormous number of patients worldwide, and hepatology is a rapidly evolving field with a great deal of newly emerging knowledge each year. The Frontiers in Medicine Research Topic “*Reviews in hepatobiliary diseases 2023*” collected seven papers, including three reviews, three meta-analyses, and a population-based study. These seven papers provide updated insights into hepatobiliary diseases.

Together with increased intestinal permeability, intestinal dysbiosis gives rise to bacterial translocation and subsequent inflammation, contributing to the progression of liver diseases thereafter. Nie et al. from China reviewed studies on the relationship between intestinal dysbiosis and liver cirrhosis. Generally, secondary to portal hypertension, abnormalities in bile acid synthesis and gut barrier injuries orchestrate the development of gut dysbiosis, while various etiologies of cirrhosis, such as viral hepatitis, alcoholic liver disease (ALD) and non-alcoholic fatty liver disease, also fuel gut dysbiosis. In addition, Nie et al. summarize the potential roles of gut dysbiosis in cirrhosis-related complications, including portal vein thrombosis (PVT), spontaneous bacterial peritonitis, hepatorenal syndrome, hepatic encephalopathy, and hepatocellular carcinoma. Therefore, the impacts of gut-derived toxins on endothelial cells, platelets or neutrophils are worthy of further study to better understand PVT and other complications.

Referring to the management of ALD, Moon et al. from Korea performed a nationwide, population-based study to evaluate the association between mean alcohol consumption and ALD risk and to determine the threshold for clinically significant alcohol consumption. The study used data from the Korean National Health Insurance database, with a large sample size of over 53,000 people collected over a period of 8 years. The results showed that drinking alcohol more than 11.5 ± 3.3 standard units/week (92 ± 26.4 g/week) significantly increased the risk of liver disease. This review offers a threshold value for alcohol consumption for the development of ALD, and it is useful for physicians to provide medical suggestions to East Asians.

In another review, Habibullah et al. from Qatar summarized the pathogenesis, diagnostic approaches and therapeutic strategies for metabolic-associated fatty liver disease (MAFLD). Multiple processes, including insulin resistance, lipotoxicity, inflammation, cytokine imbalances, activation of innate immunity, and alterations in the gut microbiota, are implicated in the progression of MAFLD. Continuing hyperglycemia causes glucotoxicity in hepatocytes, resulting in chronic inflammation, oxidative stress, endoplasmic reticulum (ER) stress, and insulin resistance. Furthermore, ER stress inhibits apoB100-mediated very low-density lipoprotein export to induce steatosis. Lipid accumulation caused by excessive lipid uptake and the upregulation of key enzymes involved in *de novo* lipogenesis results in an overload of fatty acid oxidation and reactive oxygen species production. In addition, the authors revealed associations between MAFLD and other conditions, such as chronic kidney disease, cardiovascular disease and cancer, which deteriorates the mortality of these conditions if individuals suffer from MAFLD spontaneously.

With regard to the treatment of non-alcoholic steatohepatitis (NASH), Lu et al. from China conducted a dose–response meta-analysis to explore the effect and pattern of pegbelfermin at different dosages and treatment durations on transaminase reduction in NASH patients. The currently available evidence demonstrates a positive correlation between a certain range of pegbelfermin dosages and transaminase reduction in the overall non-linear dose–response curve. This finding provides guidance for the clinical application of pegbelfermin for treating NASH.

In Tunisia, Chaouch et al. conducted a systematic review and meta-analysis to compare liver venous deprivation (LVD) with portal vein embolization (PVE) in terms of future liver remnant (FLR) volume, postoperative outcomes, and oncological safety before major hepatectomy. Compared with PVE, LVD affords patients a greater FLR volume after embolization, a greater percentage of FLR hypertrophy, less failure of resection due to a low FLR, faster kinetic growth, greater prothrombin time on day 5, and greater 3-year disease-free survival. In addition, the data for LVD are similar to those for PVE, referring to complications related to embolization, FLR percentage of hypertrophy after embolization, failure of resection, 3-month mortality, overall morbidity, major complications, operative time, blood loss, bile leakage, ascites, posthepatectomy liver failure, day 5 bilirubin level, hospital stay, and 3-year overall survival. Therefore, LVD is as promising as or even more promising than PVE for some selected patients.

With respect to pancreaticobiliary diseases, Zeng et al. reviewed the current knowledge on the treatment of sphincter of Oddi dysfunction (SOD). Previously, SOD was mainly treated surgically. However, with the development of endoscopic techniques, endoscopic intervention is currently the preferred treatment option. Endoscopic sphincterotomy (EST) is the most common endoscopic therapy for type I and type II SOD. Type I SOD patients generally benefit from EST regardless of sphincter pressure, whereas EST provides no significant benefit in type II SOD patients with normal sphincter pressure. Furthermore, a multicenter, sham-controlled, randomized trial (the EPISOD trial) demonstrated that sphincterotomy is no more effective than sham intervention for type III SOD and should not be performed in these patients. Conservative therapies, including calcium channel blockers, antispasmodics, and nitrates, have been demonstrated to be favorable for the treatment of SOD. However, most studies on this topic were uncontrolled with a weak level of evidence. It is crucial to validate the efficacy of conservative therapies in randomized controlled clinical trials.

Wang et al. performed a systematic review to compare the efficacy and safety of different advanced techniques for difficult biliary cannulation. This finding suggested that the most successful cannulation was achieved by transpancreatic sphincterotomy, followed by the double-guidewire technique and persistent standard cannulation technique. The risk of post-endoscopic retrograde cholangiopancreatography pancreatitis, overall postoperative complications, and duration of cannulation did not differ among the various techniques described above. This review could serve as a useful reference for endoscopists to make choices in patients with difficult biliary cannulation.

The articles on this Research Topic cover various aspects of hepatobiliary diseases, including liver cirrhosis, ALD, steatotic liver disease, liver surgery, SOD, and endoscopic retrograde cholangiopancreatography. The authors of each article are appreciative because their work comprehensively summarizes the updated knowledge of hepatobiliary diseases and helps us to understand this field more deeply and better.

